# Exploring yoga adherence, experiences, future preferences and barriers in the medical university community, a 6-week study

**DOI:** 10.1186/s12906-026-05268-8

**Published:** 2026-01-30

**Authors:** Fauzia Nausheen, Shazia Sheikh, Paul Lyons

**Affiliations:** California University of Science and Medicine, School of Medicine, Colton, USA

**Keywords:** Mind-body therapies, Yoga adherence, Meditation, Barriers, Expectation

## Abstract

**Background:**

Despite yoga’s known benefits for physical and mental health, studies face challenges like small samples and high attrition, especially with medical professionals showing low adherence to yoga practices.

**Objectives:**

This study aimed to assess yoga practice adherence, mainly participant experiences, future preferences, and its impact on participants’ emotional states over a 6-week period.

**Methods:**

the comprehensive yoga adherence program, including a 30-minute instructional video and weekly motivational messages and reminders. The video, accessible on various devices, was divided into pre-yoga stretches, yogic asanas, breathing exercises, and guided meditation. The study involved 15 participants from diverse backgrounds, including medical students, staff, and faculty members (above 18 years of age). Data collection was through three surveys, focusing on daily adherence, practice frequency, reasons for missed sessions, emotional states post-practice, and future yoga practice preferences. The study was conducted during the 2023/2024 academic year on the California University of Science and Medicine (CUSM) campus.

**Results:**

On average, participants engaged in yoga practice four days per week, with 17% practicing daily.: 20% of participants discontinued the practice during the study. 80% of participants reported feeling relaxed and happy after sessions, and none felt tired or bored. Common barriers to adherence included lack of time, motivation, and preference for alternative workouts. Participants expressed a strong desire to continue practicing yoga in the future, with varied preferences for virtual or in-person practice formats.

**Conclusion:**

The study offers insights into the implementation challenges and feasibility of a comprehensive yoga adherence program. It highlights factors influencing participants’ adherence and future practice preferences, contributing to effective strategies for promoting regular yoga practice and integrating it into wellness routines. The findings emphasize the need for tailored support to address diverse barriers to adherence and preferences for yoga formats. While there was significant adherence, understanding these factors is crucial for tailoring future programs to enhance participation and adherence.

**Supplementary Information:**

The online version contains supplementary material available at 10.1186/s12906-026-05268-8.

## Introduction

Yoga, an ancient spiritual practice with its roots in Indian philosophy, has gained widespread popularity in the United States, primarily as a combination of physical postures (Asanas), controlled breathing (Pranayama), and meditation (Dhyana). Recent research indicates that approximately 14% of U.S. adults have explored the practice of yoga. Among these practitioners, the most prevalent motive for yoga participation is wellness (94%), with others seeking its potential health benefits [1]. Yoga has acquired significant attention in the medical community due to its potential health benefits. Recent research by the National Institutes of Health (NIH) and the National center of Complimentary and Integrative Health NCCIH has demonstrated its positive impact on various health conditions, including stress, anxiety, depression, sleep, physical and mental balance, low-back pain, neck pain, tension-type headaches, knee osteoarthritis, weight management, menopausal symptom relief, and smoking cessation [1–5]. However, many studies have faced limitations, including small sample sizes and high attrition rates. Historically, the daily practice of yoga and meditation has been recommended for achieving optimal physical and psychological benefits, making it an integral part of a healthy lifestyle. [6] More recently, a 2025 study by Combs et al. examined adherence in a yoga intervention for veterans with chronic low back pain. While initial attendance was low (42% attending at least half the sessions), implementing strategies to improve adherence increased this to 59%. [7] Yet, the lack of adherence to regular practice and the limited support for its widespread acceptability presents challenges to studying the impact of yoga, particularly among the medical community. Despite high initiation rates of yoga and meditation practices among medical practitioner. Some studies also indicate lower attrition rates. For example, a systematic review examining yoga interventions for health professionals and students reported very low attrition rates, ranging from 0% to 26% in most included studies [8]. This suggests that yoga-based interventions may be well-tolerated and feasible within these populations’ and students, adherence remains remarkably low, with an attrition rate of nearly 90%. [9, 10] The healthcare professionals may face unique challenges in adhering to yoga practices due to demanding schedules, high stress levels, and burnout, which could potentially impact their participation rates [11]. We posit that the participation of medical students, clinicians, and faculty in yoga practices is particularly advantageous, given the high prevalence of burnout, depression, and anxiety within this demographic. [6] Yoga and mindfulness practices have the potential to create more resilient healthcare professionals and educators. Research also suggests that healthcare providers who practice yoga are more likely to recommend it to their patients based on their personal experiences and knowledge [9]. While historical barriers to yoga adherence included issues like laziness, procrastination, and physical and mental overexertion, contemporary barriers can be categorized into two main groups: strong and moderate barriers. The most suggested modern-day barriers include a lack of awareness about the benefits of yoga, unfamiliarity with appropriate yoga poses, irregular lifestyles, scheduling conflicts, lack of motivation, family commitments, work-related obligations, doubts about the efficacy of yoga, and a lack of social support. Less significant barriers include perceptions that yoga is physically demanding, preferring other forms of exercise, concerns about the quality of yoga instructors, and uncertainty about the type of yoga that suits their needs. [9–11].

Several studies have explored strategies to enhance adherence to yoga interventions. Flegal et al. (2007) found that factors such as program structure, social support, and personal motivation influenced adherence to yoga and exercise interventions in a six-month clinical trial. Bryan et al. (2012) highlighted that yoga not only improved psychosocial outcomes but also positively impacted exercise adherence, suggesting that mindfulness and stress reduction may play a role in sustaining participation. Speed-Andrews et al. (2012) identified key predictors of adherence in breast cancer survivors participating in an Iyengar yoga program, including higher baseline physical activity levels, positive attitudes toward yoga, and perceived benefits such as stress relief and improved well-being. These findings suggest that designing yoga programs with structured support, emphasizing psychological benefits, and fostering a positive participant experience can enhance long-term adherence. [12–18].

With the implementation of specific strategies, we believe that yoga adherence can be achieved among medical students, clinicians, and faculty. To develop these strategies, we conducted a pilot project of a virtual yoga program to investigate adherence to yoga and perceived barriers to daily yoga practice within our medical university community. Our study had three primary objectives:

1. Develop a user-friendly yoga program, including simple poses and guided meditation, to address barriers related to a lack of awareness about the benefits, ease of yoga poses, scheduling conflicts, and access to reliable yoga instructors.
2. Assess recruitment and attrition rates, as well as the frequency of yoga adherence and the barriers hindering consistent practice.
3. Create effective models for promoting long-term yoga adherence within the medical university community.

## Materials and methods

### Yoga program design

We designed a comprehensive yoga adherence program featuring a 30-minute video encompassing pre-yoga stretches, fundamental yogic asanas, yogic breathing exercises, and guided meditation. The 30-minute daily yoga session was chosen based on the widely recommended guideline of engaging in at least 30 min of physical activity per day for health benefits, as supported by multiple studies [19, 20] and the U.S. Department of Health and Human Services [21]. This duration ensures feasibility and adherence while aligning with general exercise recommendations.

### Yoga style

Hatha yoga was selected for this study because it is one of the most widely practiced and accessible styles, known for its balance of physical postures, breathing exercises, and relaxation techniques. Research has shown that Hatha yoga is well-tolerated and beneficial for flexibility, mindfulness, and stress reduction [22].

### Yoga protocol

The yoga protocol was designed based on recommendations from the Yoga Teacher Training at The Yoga Institute. The selected asanas focused on improving flexibility and strength, pranayama emphasized mindful breathing for stress management, and meditation aimed at enhancing relaxation and mindfulness. Previous research supports the effectiveness of these practices in promoting physical and mental well-being [23].

### Instruction and delivery

The Principal Investigator (PI), a medical doctor and certified yoga instructor, offered guidance throughout the program, delivering weekly motivational messages and reminders via email. The instructional video featured detailed guidance, with the yoga instructor leading the yoga session. The video was divided into three segments:

### Yoga segments

First 10 min: Pre-yoga stretches.

Second 10 min: Basic yogic asanas (from PI’s training at The Yoga Institute in India).

Final 10 min: Yogic breathing exercises, calming sounds, and guided meditation (informed by the PI’s training in mindfulness-based stress reduction).

The video was accessible on both phones and computers, with closed captioning and audio options available.

### Recruitment and eligibility

Recruitment procedures involved sending invitations via email and conducting live virtual or in-person meetings led by the PI/yoga teacher. These meetings explained the program, confirmed eligibility, and facilitated consent form signing.

### Inclusion criteria

Participants were required to be between 18 and 55 years old, physically fit, and without medical conditions contraindicating moderate physical activity.

### Exclusion criteria

Individuals with physical or mental conditions that could prevent safe participation in beginner-level yoga/meditation.

Eligibility was assessed through in-person discussions addressing health status, prior yoga experience, and pre-existing conditions. Informed consent was obtained from all participants.

### Study timeline

The study was conducted over six weeks during the 2023/2024 academic year on the California University of Science and Medicine (CUSM) campus at 1501 Violet St, Colton, CA 92,324.

### Data collection

Data were collected through three surveys developed for this study, administered via SurveyMonkey Enterprise to ensure accessibility and ease of response. These surveys assessed:

Daily adherence to the program.

Frequency of practice.

Reasons for missed sessions.

Post-practice emotional states.

Interest in continuing yoga practice and preferred formats (final survey).

Additional support was provided through individual check-ins conducted by the PI, aiming to address challenges and maintain engagement.

### Data analysis

Survey data were exported from SurveyMonkey into Excel spreadsheets. Built-in formulas within Excel were used for statistical computations and data analysis.

### Ethical considerations

The study received ethical approval from the Institutional Review Board (IRB) at CUSM (#HS-2023-32).

## Results

### Recruitment and retention

Out of the initial group of 20 interested candidates (above 18 years of age), 15 individuals (75%) willingly consented to participate in the study following an initial meeting with the Principal Investigator (PI). We did not record participants’ age and sex, as the surveys were anonymous. However, based on recruitment records, the study included 8 females and 6 males within the recommended age range.

### Cohort composition

The study cohort consisted of a diverse group, including 3 medical students, 6 staff members, and 6 faculty members. Following the introductory meeting, participants were introduced to yoga meditation through instructional videos, and the study duration of 6 weeks. However, during this period, 3 participants (20%) discontinued their yoga practice, resulting in an attrition rate of 20%.

### Yoga practice adherence

**Fig. 1:**
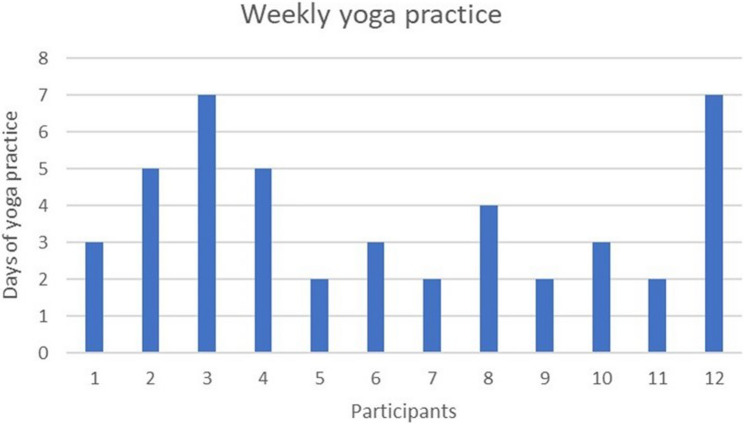
“Number of Days: The height of the bars represents the number of days each participant practiced yoga during the week.”

An analysis of adherence to yoga practice revealed several key findings: On average, participants engaged in yoga practice for four days per week. Notably, 2 participants (17%) demonstrated exceptional dedication, practicing yoga daily for all seven days of the week. (Fig. 1) Furthermore, 7 participants (58%) reported having sessions lasting more than 20 min. A majority of participants (53%) reported feeling both relaxed and happy, while 40% felt only relaxed, and 7% felt solely happy. Importantly, none of the participants reported feeling tired or bored after their practice.

### Participant-Reported outcomes of adherence

**Fig. 2:**
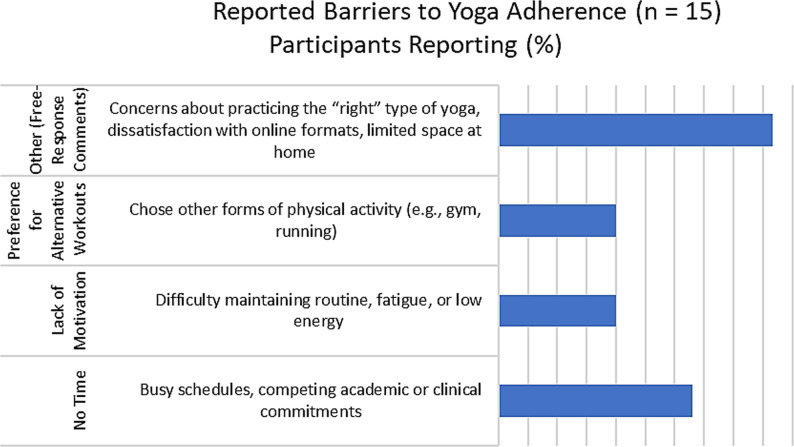
“Common Categories of Barriers to Yoga Adherence”

Among the barriers to adherence (Fig. 2), the study identified common reasons for nonadherence to daily yoga practice, with the most frequently mentioned barriers being “No Time” (33%), “Lack of Motivation” (20%), and a preference for alternative workouts (20%). Additionally, participants provided other reasons (46.6%) through free-response comments, including concerns about not practicing the right type of yoga, dissatisfaction with online or virtual practice formats, and space constraints. (Fig. 2)

Interestingly, specific trends were observed in terms of preferred days for yoga practice. Mondays were the most commonly practiced day (83%), followed by Thursdays, Fridays, and Sundays. Among the 12 participants: 25% (3 participants) preferred virtual yoga with instructional videos for individual practice. 8.3% (1 participant) preferred virtual yoga with instructional videos in a group setting. 66.7% (8 participants) favored yoga in a physical location with a yoga teacher. 33.3% (4 participants) preferred practicing independently after initial training. Additionally, 91.7% (11 participants) expressed a desire to continue practicing yoga in the future demonstrating a strong willingness to sustain their engagement with yoga.

### Discussion

Our findings shed light on the nuanced aspects of adherence, preferences, and future engagement in daily yoga practice. The flexible approach to session duration, positive emotional responses, and expressed willingness to continue yoga practice among participants suggest the potential for long-term benefits and sustained engagement in yoga and meditation. Among a diverse cohort of 20 interested participants, 15 (75%) participants consented to commit to daily yoga practice. Notably, our attrition rate of 20% stood in contrast to previous studies reporting attrition rates exceeding 80%, particularly among medical students. [9, 10] Analyzing adherence patterns, we observed that participants, on average, engaged in yoga practice 4–5 days per week, with a noteworthy 17% maintaining daily practice. Despite the recommended 30-minute sessions by the principal investigator (PI), participants reported engaging in sessions that averaged around 20 min, with a notable variability spanning 10 to 30 min. This outcome suggests that a flexible approach to the duration of practice sessions may contribute significantly to sustaining participant engagement. The emotional responses post-practice was overwhelmingly positive, with 53% of participants reporting feeling both relaxed and happy. This aligns with trends observed in many yoga studios, where Mondays tend to draw the maximum attendance. [23–27]. Importantly none of the participants reported feelings of tiredness or boredom, emphasizing a positive emotional response to yoga and meditation, as noted in related research.

Participants demonstrated intriguing preferences for specific days, with Monday being the most popular choice (83%).23 This aligns with trends observed in many yoga studios, where Mondays tend to draw the maximum attendance. The study also uncovered diverse format preferences, ranging from virtual sessions with instructional videos to in-person classes or individual practice. This divergence in preferences echoes the broader choices made by yoga practitioners, some favoring group dynamics, while others prefer solitary practice after receiving initial training. Encouragingly, 11 out of 12 participants expressed a desire to continue practicing yoga in the future, indicating a positive outlook and potential for sustained engagement. This aligns with previous studies emphasizing the longevity of engagement in yoga meditation.28.29.30 Barriers to adherence revealed common challenges, with "No Time" being the most frequently mentioned obstacle, consistent with broader survey studies highlighting time constraints as a hindrance to yoga practice. To address motivation and time constraints, we included weekly motivational messages emphasizing the spiritual and emotional benefits of yoga over alternative workouts. However, "Lack of Motivation" (20%) and a preference for alternative workouts (20%) persisted, despite attempts to mitigate these issues. [31–33].

This study underscored the significance of personalized attention, highlighting the role of regular check-ins conducted by the yoga teacher or program instructor in maintaining participant engagement. Such practices are essential to guarantee that participants receive individualized guidance and support specifically tailored to their unique needs. [34–36]. Furthermore, encouragement from instructors or peers has proven to be a valuable motivational throughout the practice journey. [37, 38].

We did not collect specific data on how many participants divided their yoga practice between morning and pre-bedtime sessions. Based on our informal conversation, we suggest a flexible approach to yoga practice by dividing it into two distinct parts: morning stretches and pre-bedtime meditation. This structure includes specific asanas for morning flexibility and meditation techniques for relaxation before sleep. The goal of this two-step approach is to enhance participant engagement in yoga programs by making practice more adaptable to daily routines. Notably, we did not provide a diverse range of yoga styles, which may have influenced yoga adherence. Offering a variety of yoga styles would have empowered participants to make informed choices based on their preferences and needs. [39, 40].

**Fig. 3:**
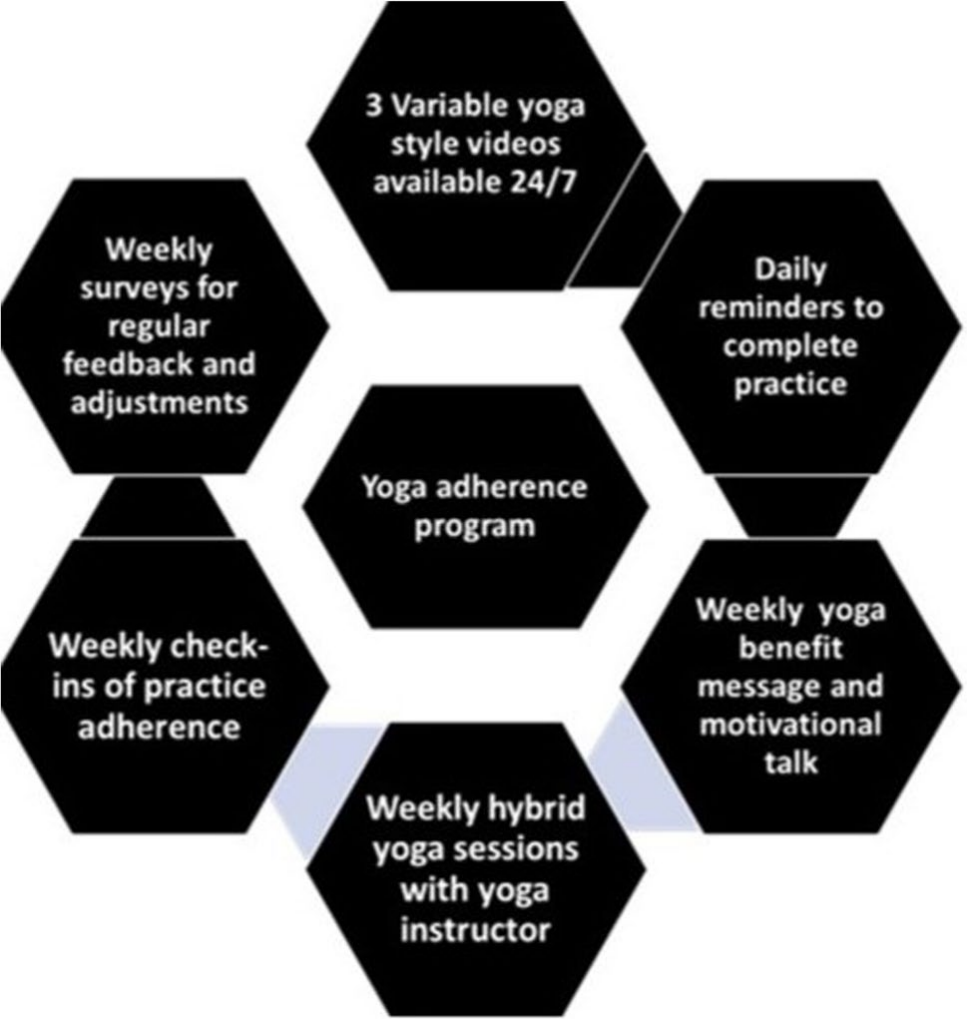
“Components of Yoga Adherence Program: Each hexagon represents a different aspect of the yoga adherence program. All components are integral to the support system designed to encourage regular yoga practice.”

These findings offer valuable insights into the factors influencing adherence to yoga practice and highlight the potential feelings of yoga practice for promoting relaxation and positive emotions.41,42 Understanding the barriers and preferences of participants can guide the development of tailored yoga programs to enhance engagement and promote overall well-being. Based on the insights gained from this study, future strategies for enhancing adherence to yoga programs can be designed to address identified barriers and capitalize on positive participant experiences. Here are some key strategies that can be implemented for future yoga adherence program: (Fig 3).

The findings from our study provide strong evidence for the need to implement flexible, engaging, and personalized yoga practice strategies to enhance adherence and long-term engagement. Key insights from the study data support the following future program initiatives:

### Flexible scheduling solutions

The study identified "No Time" as the most frequently cited barrier to daily yoga practice, affecting 33% of participants. This highlights the necessity of introducing flexible scheduling solutions, including video-based practices available at different times (early morning, afternoon, or before bed). Furthermore, dividing the practice into two shorter sessions aligns with participants’ needs, allowing those with time constraints to maintain consistency.

### Varied practice durations and formats

The study revealed that 58% of participants engaged in yoga sessions lasting more than 20 minutes. However, given that "No Time" remains a key barrier, offering varied practice durations (15-30 minutes) will cater to diverse schedules and encourage greater participation.

These findings offer valuable insights into the factors influencing adherence to yoga practice and highlight the potential feelings of yoga practice for promoting relaxation and positive emotions [41, 42]. Understanding the barriers and preferences of participants can guide the development of tailored yoga programs to enhance engagement and promote overall well-being.

The findings from our study provide strong evidence for the need to implement flexible, engaging, and personalized yoga practice strategies to enhance adherence and long-term engagement. Key insights from the study data support the following future program initiatives:

### Flexible scheduling solutions

The study identified “No Time” as the most frequently cited barrier to daily yoga practice, affecting 33% of participants. This highlights the necessity of introducing flexible scheduling solutions, including video-based practices available at different times (early morning, afternoon, or before bed). Furthermore, dividing the practice into two shorter sessions aligns with participants’ needs, allowing those with time constraints to maintain consistency.

### Varied practice durations and formats

The study revealed that 58% of participants engaged in yoga sessions lasting more than 20 min. However, given that “No Time” remains a key barrier, offering varied practice durations (15–30 min) will cater to diverse schedules and encourage greater participation.

### Continuous support and community engagement

Despite the initial engagement of 15 participants, a 20% attrition rate was observed. However, 91.7% of participants expressed a desire to continue practicing yoga in the future, indicating that additional support mechanisms such as check-ins, follow-ups, and virtual communities could enhance adherence and long-term commitment.

### Enhanced motivational strategies

“Lack of Motivation” was reported as a barrier by 20% of participants. To address this, incorporating personal goal setting, progress tracking, and mindfulness techniques will be instrumental in sustaining enthusiasm and commitment.

### Personalized guidance and support

The study revealed a preference for structured guidance, with 66.7% of participants favoring in-person yoga sessions with a teacher. Additionally, 33.3% preferred independent practice after initial training. This underscores the importance of continued personalized guidance through regular instructor check-ins to cater to individual needs.

### Customized practice plans

Some participants cited concerns about “not practicing the right type of yoga,” indicating a need for tailored approaches. Offering customized practice plans, either through instructor collaboration or online tools, will help participants align their yoga practice with personal goals and preferences.

### Online and in-person hybrid options

A significant portion of participants (66.7%) preferred in-person yoga instruction, whereas 25% found virtual yoga with instructional videos suitable for individual practice. Introducing hybrid options, combining both virtual and in-person sessions, will accommodate diverse preferences.

### Goal setting for long-term engagement

With 91.7% of participants expressing a willingness to continue practicing yoga, encouraging long-term goal setting will further reinforce yoga as a sustainable wellness practice.

### Highlighting emotional benefits

A majority of participants (53%) reported feeling both relaxed and happy, while 40% felt only relaxed after practice. None reported feeling tired or bored. Emphasizing these emotional benefits in future programs will help motivate continued engagement.

### Continuous feedback mechanisms

The study’s free-response data revealed concerns about yoga style suitability, dissatisfaction with virtual formats, and space constraints. Incorporating regular feedback mechanisms will allow for ongoing improvements, ensuring programs remain relevant and effective.

### Coping strategies for life events

Given that 20% of participants discontinued their yoga practice during the study period, integrating coping strategies for life transitions will provide essential tools to maintain consistency in challenging times.

### Emphasis on supportive community

Peer encouragement and instructor reinforcement were shown to be key factors in sustaining motivation. Maintaining a strong, supportive community will be critical for long-term engagement.

### Availability of a variety of yoga styles

Concerns about yoga type suitability suggest that offering a range of yoga styles, along with trial sessions and workshops, will help participants make informed choices and increase adherence.

In summary, our commitment to providing holistic support remains steadfast. By implementing these future strategies, we aspire to create an environment where participants feel empowered, motivated, and connected throughout their yoga practice journey.

### Limitations

While this study provides valuable insights into yoga adherence and barriers within the medical university community, it’s essential to acknowledge its limitations to be taken into consideration for planning the future research endeavors on yoga and meditation:

### Small sample size and short duration

We employed a convenience sampling approach, and the relatively small sample size may limit the generalizability of our findings to broader populations. This study was designed as a pilot project intended to generate preliminary insights and inform the development of larger, controlled trials. The duration was 6 weeks, which may not have revealed all potential barriers to yoga adherence. We acknowledge the potential for observer bias since the PI was also the yoga instructor in the video; however, we have outlined the steps taken to mitigate this risk, including standardized delivery of content and use of objective outcome measures.

## Conclusion

These findings shed light on the nuances of yoga adherence and provide valuable insights into participants’ barriers and preferences, offering guidance for developing future yoga programs tailored to individual needs and circumstances. Future strategies should emphasize flexibility, motivation, and adaptability to support sustained engagement. By addressing identified barriers, instructor time, space, sustainability and reinforcing the positive aspects of participants’ experiences, institutional yoga programs can foster long-term participation, enhance well-being, and promote sustainable wellness practices within academic communities.

## Supplementary Information

Supplementary Material 1.

## Data Availability

The datasets used and/or analyzed during the current study are available from the corresponding author upon reasonable request.

## Abbreviations

Abbreviation: Definition
Asanas: Physical postures in yoga practice
CUSM: California University of Science and Medicine
Dhyana: Meditation in yoga practice
IRB: Institutional Review Board
NCCIH: National Center for Complementary and Integrative Health
NIH: National Institutes of Health
PI: Principal Investigator
Pranayama: Controlled breathing techniques in yoga
FN: Fauzia Nausheen
SS: [Co-author initials, e.g., co-author name
PL: [Co-author initials, e.g., co-author name
AR: Attrition Rate
ADH: Adherence
VY: Virtual Yoga (yoga with instructional videos)
IPY: In-Person Yoga (yoga with a teacher physically present)
INDY: Independent Practice (participant practices yoga individually after initial training)
YP: Yoga Practice
EM: Emotional Response (after yoga practice)
MT: Motivation (as a barrier to yoga adherence)
NT: No Time (as a barrier to yoga adherence)
WP: Willingness to Continue Practice

## References

[1] National Center for Complementary and Integrative Health. Yoga: What you need to know. 2024. Available from: https://www.nccih.nih.gov/health/yoga-what-you-need-to-know. [cited 2025 Feb 28].

[2] Anheyer D, Klose P, Lauche R, Saha FJ, et al. Yoga for treating headaches: A systematic review and meta-analysis. J Gen Intern Med. 2020;35(3):846–54. 10.1007/s11606-019-05413-9. 31667736 10.1007/s11606-019-05413-9 PMC7080891

[3] Batrakoulis A. Psychophysiological adaptations to yoga practice in overweight and obese individuals: A topical review. Diseases. 2022;10(4):107. 10.3390/diseases10040107. 36412601 10.3390/diseases10040107 PMC9680480

[4] Black LI, Barnes PM, Clarke TC, Stussman BJ, Nahin RL. Use of yoga, meditation, and chiropractors among U.S. Children aged 4–17 years. NCHS Data Brief. 2018;(324):1–8. 30475687

[5] Bock BC, Dunsiger SI, Rosen RK, et al. Yoga as a complementary therapy for smoking cessation: results from BreathEasy, a randomized clinical trial. Nicotine Tob Res. 2019;21(11):1517–23. 10.1093/ntr/nty212. 30295912 10.1093/ntr/nty212 PMC6821291

[6] What happens to your body when you do yoga every day?. Verywell Fit. 2025. Available from: https://www.verywellfit.com/daily-yoga-7511375 [cited 2025 Feb 28].

[7] Gonzalez C, Chang DG, Rutledge T, Groessl EJ. Promoting adherence to a yoga intervention for veterans with chronic low back pain. Glob Adv Integr Med Health. 2025;14:27536130251323247. 10.1177/27536130251323247. 39989733 10.1177/27536130251323247 PMC11846116

[8] Ciezar-Andersen SD, Hayden KA, King-Shier KM. A systematic review of yoga interventions for helping health professionals and students. Complement Ther Med. 2021;58:102704. 10.1016/j.ctim.2021.102704. 33652090 10.1016/j.ctim.2021.102704

[9] Angadi P, Jagannathan A, Thulasi A, Kumar V, Umamaheshwar K, Raghuram N. Adherence to yoga and its resultant effects on blood glucose in type 2 diabetes: A community-based follow-up study. Int J Yoga. 2017;10(1):29–36. 28149065 10.4103/0973-6131.186159 PMC5225741

[10] Brems C, Justice L, Sulenes K, et al. Improving access to yoga: barriers to and motivators for practice among health professions students. Adv Mind Body Med. 2015;29(3):6–13. 26026151

[11] Flegal KE, Kishiyama S, Zajdel D, Haas M, Oken BS. Adherence to yoga and exercise interventions in a 6-month clinical trial. BMC Complement Altern Med. 2007;7:1–7. 17996075 10.1186/1472-6882-7-37 PMC2194735

[12] Bryan S, Zipp GP, Parasher R. The effects of yoga on psychosocial variables and exercise adherence: A randomized, controlled pilot study. Altern Ther Health Med. 2012;18(5). https://pubmed.ncbi.nlm.nih.gov/22894891/. 22894891

[13] Speed-Andrews AE, Stevinson C, Belanger LJ, Mirus JJ, Courneya KS. Predictors of adherence to an Iyengar yoga program in breast cancer survivors. Int J Yoga. 2012;5(1):3–9. 22346059 10.4103/0973-6131.91693 PMC3276930

[14] Hegde SV, Rao SK, Menezes RG, et al. Knowledge, attitude, and practice of yoga in medical students: assessment of anthropometry and lifestyle factors. Int J Yoga Ther. 2018;28(1):9–14. 10.17761/2018-00005R1. 10.17761/2018-00005R1 29596004

[15] Appiani FJ, Rodríguez Cairoli F, Sarotto L, et al. Prevalence of stress, burnout syndrome, anxiety and depression among physicians of a teaching hospital during the COVID-19 pandemic. Arch Argent Pediatr. 2021;119(5):317–24. 10.5546/aap.2021.eng.317. 34569739 10.5546/aap.2021.eng.317

[16] Baspure S, Jagannathan A, Kumar S, et al. Barriers to yoga therapy as an add-on treatment for schizophrenia in India. Int J Yoga. 2012;5(1):70–3. 10.4103/0973-6131.91718. 22346070 10.4103/0973-6131.91718 PMC3276937

[17] Spadola CE, Rottapel R, Khandpur N, et al. Enhancing yoga participation: A qualitative investigation of barriers and facilitators to yoga among predominantly racial/ethnic minority, low-income adults. Complement Ther Clin Pract. 2017;29:97–104. 10.1016/j.ctcp.2017.09.001. 29122272 10.1016/j.ctcp.2017.09.001 PMC5786160

[18] Dayananda H, Ilavarasu JV, Rajesh S, Babu N. Barriers in the path of yoga practice: an online survey. Int J Yoga. 2014;7(1):66–71. 10.4103/0973-6131.123490. 25035610 10.4103/0973-6131.123490 PMC4097919

[19] Warburton DER, Bredin SSD. Health benefits of physical activity: A systematic review of current systematic reviews. Curr Opin Cardiol. 2017;32(5):541–56. 10.1097/HCO.0000000000000437. 28708630 10.1097/HCO.0000000000000437

[20] Ross A, Thomas S. The health benefits of yoga and exercise: A review of comparison studies. J Altern Complement Med. 2010;16(1):3–12. 10.1089/acm.2009.0044. 20105062 10.1089/acm.2009.0044

[21] Department of Health &. Human Services. HHS.gov. Available from: https://www.hhs.gov. 10.3109/15360288.2015.1037530 26095483

[22] Cramer H, Lauche R, Anheyer D, Pilkington K, de Manincor M, Dobos G, Ward L. Yoga for anxiety: A systematic review and meta-analysis of randomized controlled trials. Depress Anxiety. 2018;35(9):830–43. 10.1002/da.22762. 29697885 10.1002/da.22762

[23] Saltonstall E, Fishman L. Yoga for arthritis. New York: W. W. Norton & Company.

[24] Gorvine MM, Haynes TF, Marshall SA, et al. An exploratory study of the acceptability and feasibility of yoga among women in substance use disorder recovery. J Altern Complement Med. 2021;27(3):273–81. 10.1089/acm.2020.0351. 33373528 10.1089/acm.2020.0351 PMC7989855

[25] Prathikanti S, Rivera R, Cochran A, et al. Treating major depression with yoga: A prospective, randomized, controlled pilot trial. PLoS ONE. 2017;12(3):e0173869. 10.1371/journal.pone.0173869. 28301561 10.1371/journal.pone.0173869 PMC5354384

[26] Burnett-Zeigler I, Zhou E, Martinez JH, et al. Comparative effectiveness of a mindfulness-based intervention (M-Body) on depressive symptoms: study protocol of a randomized controlled trial in a federally qualified health center (FQHC). Trials. 2023;24(1):115. 10.1186/s13063-022-07012-2. 36803835 10.1186/s13063-022-07012-2 PMC9936464

[27] Hagen I, Skjelstad S, Nayar US. Promoting mental health and wellbeing in schools: the impact of yoga on young people’s relaxation and stress levels. Front Psychol. 2023;14:1083028. 10.3389/fpsyg.2023.1083028. 37265958 10.3389/fpsyg.2023.1083028 PMC10229855

[28] Cheung C, Justice C, Peden-McAlpine C. Yoga adherence in older women six months post-osteoarthritis intervention. Glob Adv Health Med. 2015;4(3):16–23. 10.7453/gahmj.2015.041. 25984414 10.7453/gahmj.2015.041 PMC4424934

[29] Gothe NP, Khan I, Hayes J, et al. Yoga effects on brain health: A systematic review of the current literature. Brain Plast. 2019;5(1):105–22. 10.3233/BPL-190084. 31970064 10.3233/BPL-190084 PMC6971819

[30] Moszeik EN, von Oertzen T, Renner KH. Effectiveness of a short yoga Nidra meditation on stress, sleep, and well-being in a large and diverse sample. Curr Psychol. 2022;41:5272–86. 10.1007/s12144-020-01042-2.

[31] Woodyard C. Exploring the therapeutic effects of yoga and its ability to increase quality of life. Int J Yoga. 2011;4(2):49–54. 10.4103/0973-6131.85485. 22022122 10.4103/0973-6131.85485 PMC3193654

[32] Polsgrove MJ, Eggleston BM, Lockyer RJ. Impact of 10-weeks of yoga practice on flexibility and balance of college athletes. Int J Yoga. 2016;9(1):27–34. 10.4103/0973-6131.171710. 26865768 10.4103/0973-6131.171710 PMC4728955

[33] Naragatti S. The study of yoga effects on health. Yoga J. 2020;98:110.

[34] Tamminga SJ, Emal LM, Boschman JS, et al. Individual-level interventions for reducing occupational stress in healthcare workers. Cochrane Database Syst Rev. 2023;5(5):CD002892. 10.1002/14651858.CD002892.pub6. 37169364 10.1002/14651858.CD002892.pub6 PMC10175042

[35] Nalbant G, Lewis S, Chattopadhyay K. Characteristics of yoga providers and their sessions and attendees in the UK: A cross-sectional survey. Int J Environ Res Public Health. 2022;19(4):2212. 10.3390/ijerph19042212. 35206399 10.3390/ijerph19042212 PMC8871723

[36] Telles S, Sharma SK, Gupta RK, et al. The impact of yoga on teachers’ self-rated emotions. BMC Res Notes. 2019;12:680. 10.1186/s13104-019-4737-7. 31640779 10.1186/s13104-019-4737-7 PMC6805642

[37] Law BM, Siu AM, Shek DT. Recognition for positive behavior as a critical youth development construct: conceptual bases and implications on youth service development. Sci World J. 2012;2012:809578. 10.1100/2012/809578. 10.1100/2012/809578 PMC3361320 22666155

[38] Vo TTD, Tuliao KV, Chen CW. Work motivation: the roles of individual needs and social conditions. Behav Sci (Basel). 2022;12(2):49. 10.3390/bs12020049. 35200300 10.3390/bs12020049 PMC8869198

[39] Cocchiara RA, Peruzzo M, Mannocci A, et al. The use of yoga to manage stress and burnout in healthcare workers: A systematic review. J Clin Med. 2019;8(3):284. 10.3390/jcm8030284. 30813641 10.3390/jcm8030284 PMC6462946

[40] Harden SM, Steketee AM, Kelliher R, et al. Using a studio-academic partnership to advance public health within a pragmatic yoga setting. J Prim Care Community Health. 2019;10:2150132719874621. 10.1177/2150132719874621. 31538842 10.1177/2150132719874621 PMC6755627

[41] Shohani M, Badfar G, Nasirkandy MP, et al. The effect of yoga on stress, anxiety, and depression in women. Int J Prev Med. 2018;9:21. 10.4103/ijpvm.IJPVM_242_16. 29541436 10.4103/ijpvm.IJPVM_242_16 PMC5843960

[42] Dike IC, Onyishi CN, Adimora DE, et al. Yoga complemented cognitive behavioral therapy on job burnout among teachers of children with autism spectrum disorders. Med (Baltim). 2021;100(22):e25801. 10.1097/MD.0000000000025801. 10.1097/MD.0000000000025801 PMC8183729 34087823

