# Supplementary material for: Exploring yoga adherence, experiences, future preferences and barriers in the medical university community, a 6-week study
Source: BMC Complement Med Ther. 2026 Jan 30;26:73. doi: 10.1186/s12906-026-05268-8 (PMC12930759; doi:10.1186/s12906-026-05268-8)
Supplement: Supplementary file 1 — Supplementary Material 1. [file 12906_2026_5268_MOESM1_ESM.docx]

**Protocol: 30-Minute Hatha Yoga Session**

The yoga session begins with a brief centering and warm-up (wrist, elbow, neck, and leg mobilization, ~5 minutes). This is followed by standing postures including forward bend, one-legged stand, triangle, and palm tree (~8 minutes). Floor-based postures include child’s pose, cobra, downward dog, lying leg bends, and shoulder stand (~8 minutes). The session transitions to relaxation in Savasana (~2 minutes), then seated practices: kapalabhati breathing, alternate nostril breathing (~3 minutes), and concludes with full-body relaxation and meditation (~4 minutes). Total time: ~30 minutes.

**Justification**

This structured Hatha Yoga protocol balances gentle joint mobilization, standing poses for strength and balance, floor postures for flexibility and circulation, and breathing practices for autonomic regulation. The inclusion of Savasana and meditation supports mind-body relaxation and stress reduction. Such integrative yoga sessions have been shown to improve physical fitness, enhance psychological well-being, and support stress management in diverse populations (Field, 2016; Woodyard, 2011).

**Reference:**

- Woodyard C. (2011). Exploring the therapeutic effects of yoga and its ability to increase quality of life. *International Journal of Yoga*, 4(2), 49–54.
- Field T. (2016). Yoga research review. *Complementary Therapies in Clinical Practice*, 24, 145–161.

**30-Minute Hatha Yoga Script**

Opening (2 min)

- Sit comfortably, cross-legged.
- Close eyes, hands on knees.
- Take 3 deep breaths in and out, grounding into the practice.

Gentle Warm-Up (5 min)

1. Wrist rotations – rotate both wrists clockwise and counterclockwise (1 min).
2. Elbow rotations – gentle circles forward and back (1 min).
3. Neck stretches – slow side to side, forward and back, gentle circles (2 min).
4. Leg movements – gentle knee lifts and ankle circles (1 min).

Standing Asanas (8 min)

1. Forward Bend (Uttanasana) – Fold forward, soften knees, relax head and arms (1 min).
2. One-Legged Stand (Eka Pada Pradasana) – Balance on one foot, lift opposite leg, switch sides (1 min each).
3. Triangle Pose (Trikonasana) – Extend arms, bend to side, switch sides (2 min total).
4. Palm Tree Pose (Talasana) – Inhale, stretch arms overhead, rise on toes, lengthen spine (1 min).

Floor Asanas (8 min)

1. Child Pose (Balasana) – Kneel, fold forward, arms extended (1 min).
2. Cobra (Bhujangasana) – Lie prone, lift chest with inhalation (1 min).
3. Downward Dog (Adho Mukha Svanasana) – Lift hips, lengthen spine, press heels (2 min).
4. Lying Leg Bending (Pawanmuktasana) – Hug one knee at a time, then both knees to chest (2 min).
5. Shoulder Stand (Sarvangasana) – Lie back, lift legs and torso, support lower back with hands (2 min).

Relaxation & Breathing (6 min)

1. Corpse Pose (Savasana) – Lie flat, arms open, eyes closed, breathe naturally (2 min).
2. Seated, Cross-Legged – Transition gently to sitting (30 sec).
   - Kapalabhati – Rapid exhalations with passive inhalation (30 sec × 2 rounds).
   - Alternate Nostril Breathing (Nadi Shodhana) – Inhale left, exhale right; inhale right, exhale left (2 min).
3. Full Body Relaxation / Short Meditation – Observe breath, body awareness, quiet mind (1.5 min).

Closing (1 min)

- Sit with palms together at heart center.
- Take a deep breath, exhale slowly, and bow the head with gratitude.

Total ≈ 30 minutes
